# Electrical and Functional Magnetic Stimulation in the Management of Children’s Lower Urinary Tract Dysfunction—A Current Literature Review

**DOI:** 10.3390/children13030322

**Published:** 2026-02-25

**Authors:** Edva Anna Frunda, András Kiss, Árpád Olivér Vida, Tibor Lóránd Reman, Raul-Dumitru Gherasim, Daniel Porav-Hodade, Virgil Gheorghe Osan, Carmen Viorica Muntean, Orsolya Katalin Ilona Mártha, Lorena Meliț

**Affiliations:** 1 Institution Organizing University Doctoral Studies (I.O.S.U.D.), George Emil Palade University of Medicine, Pharmacy, Science and Technology of Targu Mures, 540139 Targu Mures, Romania; frunda.anna@gmail.com (E.A.F.);; 2 Department of Pediatrics–Heim Pál, Semmelweis University Budapest, Baross utca 22, 1085 Budapest, Hungary; kiss.andras@med.semmelweis-univ.hu; 3 Department of Urology, George Emil Palade University of Medicine, Pharmacy, Science, and Technology of Targu Mures, 540139 Targu Mures, Romania; 4 Department of Pediatrics, George Emil Palade University of Medicine, Pharmacy, Science, and Technology of Targu Mures, 540139 Targu Mures, Romania; carmen.duicu@umfst.ro; 5 Department of Pediatrics, County Emergency Clinical Hospital of Targu Mures, Str. Gh. Marinescu Nr.50, 540136 Targu Mures, Romania; 6 Department of Pediatrics II, George Emil Palade University of Medicine, Pharmacy, Science, and Technology of Targu Mures, 540139 Targu Mures, Romania

**Keywords:** functional magnetic stimulation, electrical stimulation, children’s lower urinary tract dysfunction, children’s overactive bladder, enuresis

## Abstract

Background and Objective: Children’s lower urinary tract dysfunction (LUTD) is a frequently diagnosed condition. Initial management measures, such as parental education, urotherapy, and constipation management, followed by drug-based and surgical therapies, are well known. In recent years, different electrical, transcutaneous nerve stimulation methods (sacral, tibial, and intravesical) were studied, and magnetic stimulation has gained importance in the treatment of lower urinary tract dysfunctions. This review aimed to examine the efficacy of functional magnetic stimulation in treating children’s LUTD. Materials and Methods: Our review was carried out in the following databases: PubMed/Medline and Google Scholar. Results: We limited our search to articles published in the last 8 years (2018–2025). Our used keywords were: “functional magnetic stimulation, electrical stimulation, children’s lower urinary tract dysfunction, children’s overactive bladder, enuresis”. The final search revealed a total of 132 results; articles written in languages other than English were excluded. In the end, a total of 16 articles presenting ES (12 articles) and FMS (four articles) in the treatment of children’s lower urinary tract dysfunction were included in our narrative review. Conclusions: In spite of a few clinical controlled trials, our review concerning functional magnetic stimulation (FMS) provides acceptable evidence to support the efficacy of these methods among the pediatric population. In the future, large sample, randomized controlled trials are needed in this field.

## 1. Introduction

Lower urinary dysfunction (dysfunctional micturition) symptoms are frequently encountered during childhood, defining the bladder storage and/or voiding phase of bladder function. Moreover, urinary tract infections, urinary incontinence (stress, urge, or mixed), overactive bladder, and vesicoureteral reflux are highly prevalent in children, triggering stress and anxiety. This broad spectrum of lower urinary symptoms can be accompanied by bowel disturbances and dysfunctions (constipation, encopresis, etc.), labeled as bladder bowel dysfunction (BBD). Other important key factors that contribute to micturition and defecation are represented by the prostate in men, the uterus in women, the rectum, and the pelvic floor muscles [1].

The management of children’s LUTD is very challenging due to both their vulnerability and the lifelong implications of these disorders. Therefore, patients diagnosed with this condition deserve special attention and close monitoring. The evolution of the aforementioned disorder as a result of a lack of appropriate treatment may lead to progressive upper tract deterioration and chronic renal failure. Several urological procedures are performed during the evolution of urinary tract dysfunction, aiming to preserve the filling and voiding functions of the urinary tract, among which are non-invasive procedures (parental education, bladder diary, and constipation management) and urotherapy [1].

The rehabilitation strategies meant to preserve a normal voiding function include pelvic floor muscle exercises, biofeedback, electrical stimulation, neuromodulation, and clean intermittent self-catheterization [2]. Electrical stimulation is a procedure that not only improves, but can also cure, various micturition symptoms, such as urgency and frequency, by enhancing the urethral closure, inhibiting or reducing bladder instability, and maintaining the good function of the pelvic muscles. Bladder electro-neurostimulation seems to be a good alternative in the management of children’s voiding dysfunctions [1,2]. Recently, functional magnetic stimulation has gained attention, given its good results in the pediatric population. This novel approach proved to be effective in various micturition disorders, such as pediatric nocturnal enuresis, but also urinary incontinence, and chronic pelvic pain syndrome, regardless of age and gender [3]. Magnetic stimulation therapy is a method initially used in neurology and orthopedics, which has proven its efficacy in a broad spectrum of pelvic floor organs and muscle disorders [1,2].

## 2. Materials and Methods

Considering the promising outcomes of electro-neurostimulation, magnetic therapy is expected to further develop as a new therapeutic modality. To establish its role and results in the pediatric population, we performed a literature search using PubMed/Medline and Google Scholar databases. We limited our search to articles published in the last 8 years (2018–2025). Our used keywords were: “functional magnetic stimulation, electrical stimulation, children’s lower urinary tract dysfunction, children’s overactive bladder, enuresis”. The final search revealed a total of 132 results. Various research reports have been published on the therapeutic effect of magnetic stimulation in the case of adults; articles written in languages other than English were excluded. In the end, a total of 16 articles presenting ES (12 articles) and FMS (four articles) in the treatment of children’s dysfunctional voiding were included in our narrative review.

## 3. Results

Despite the large number of non-invasive therapeutic procedures available for children’s micturition disorders, electrical stimulation, involving the recently reported extracorporeal electro-magnetic stimulation therapy, might be an effective option for these cases.

### 3.1. Electrostimulation

Electrical stimulation, specifically sacral, parasacral transcutaneous electrical nerve stimulation (TENS), has been reported by current studies to be an alternative treatment for pediatric overactive bladder syndrome. Thus, a prospective multicenter study involving patients with overactive bladder (OAB) refractory to anticholinergics (ACHS) assessed the effect of sacral-TENS (S-TENS) administered for more than 3 months and concluded that it is a safe and effective treatment option for patients diagnosed with OAB refractory to ACHS. Nevertheless, the authors noticed that the effects of S-TENS did not improve the long-term evolution of their studied population. Moreover, the progression of symptoms reported by the patients included in the study was assessed using a voiding diary and the Pediatric Lower Urinary Tract Symptoms Score (PLUTSS), and enuresis items were removed. An additional important finding of the aforementioned study was the low rate of complications, of only 3%, involving local dermatitis of the stimulation area [3]. Similar findings were also reported by an international, multicenter, prospective study involving pediatric patients aged between 6 and 16 years (28 girls and 25 boys), diagnosed with overactive bladder (OAB) in two centers from Australia, one from Germany, and one from Brazil, which found an improvement in urinary symptoms and QoL, but no change in psychological symptoms. It is worth mentioning that in order to improve the power of the study, the authors excluded from their study children with anatomical and/or neurological anomalies affecting the urinary tract [4]. Moreover, a prospective randomized study including 86 children with no resolution of the symptoms after standard urotherapy and constipation management, proved that electrotherapy is more effective than oxybutynin, having a higher rate of complete clinical resolution and a significantly lower incidence of adverse effects, among which local dermatitis, fecal losses, and urgency are reported as possible side effects for electrotherapy [5]. Maternik M. et al. assessed the impact of parasacral-TENS in 57 children with OAB, with a mean age of 10.8 years, and noticed that this type of treatment reduced daytime incontinence, nocturnal enuresis, and all urgency episodes. According to this study, an improvement in urinary symptoms was reported after a period of four months of continuous treatment and lasted for six months after treatment cessation [6]. Another prospective study performed by Cassal Beloy et al. aimed to assess the efficacy and safety of home-based TENS in 21 children, aged between 6 and 16 years, presenting symptoms like incontinence (89%) and urgency (100%). After 6 months of electrostimulation, the authors highlighted that home-based TENS is an effective and safe option for OAB in the pediatric population; however, further randomized studies are needed to standardize all treatment protocols and to better clarify the effectiveness of this therapeutic approach [7].

A prospective randomized study evaluated biofeedback and parasacral electric nerve stimulation in 64 children (mean age 9.39) manifesting lower urinary tract dysfunction, who were divided into two groups regardless of the predominant treatment type of voiding dysfunctions. The initial assessment consisted of history, physical examination, urinalysis, a voiding diary, ultrasound, and uroflowmetry. Both methods demonstrated similar efficacy. However, in terms of biofeedback, fewer sessions were necessary in order to achieve the same good clinical results and in parasacral electric nerve stimulation [8]. A randomized controlled trial conducted by Liu Y. performed on 83 children with neurogenic OAB non-responsive to ACHS treatment also assessed the role of transcutaneous electrical nerve stimulation. In this study, the patients were divided into two groups: the transcutaneous electrical nerve stimulation group (30 min, once a day for 90 days) and the control group. The authors reported a cessation of ACHS treatment in patients included in the transcutaneous electrical nerve stimulation when compared to the control group. Thus, the authors were entitled to conclude that transcutaneous electrical stimulation over the sacral region for 90 days improved both overactive bladder symptoms and urodynamic values [9]. A recent meta-analysis summarized that electrotherapy appears to improve bladder compliance, cystometric capacity, and detrusor pressure in children with neurogenic bladder due to spina bifida. However, due to the variability in study quality and intervention parameters, further high-quality research is needed to confirm its clinical efficacy [10].

Parasacral TENS (P-TENS) also proved to be effective in other LUTDs of childhood, such as mono-symptomatic nocturnal enuresis in children and adolescents. Thus, four randomized clinical trials involving 146 children and adolescents aged between 6 and 16.3 years with nocturnal enuresis—who were administered 20 min sessions of parasacral transcutaneous electrical nerve stimulation, 3 times a week, with a frequency of 10 Hz and a pulse duration of 700 µs—confirmed that that P-TENS reduces wet night’s frequency but does not cure them [11].

Kırlı EA et al. performed a single-center study on 12 children (boys: 8, girls: four) diagnosed with overactive bladder refractory to standard treatment protocol and medication, and underlined that P-TENS has emerged as a therapeutic alternative. In the study of Kırlı EA, P-TENS was administered over the S2-S3 dermatomes over a 3-month period, twice a week, and each session lasted for 20 min, and used a frequency of 10 Hz and a pulse duration of 250 μs. The evaluation at 6 months after the last procedure revealed significant improvement in uroflow parameters. Additionally, not only were the urge incontinence (*p* = 0.016) and constipation (*p* = 0.031) rates significantly decreased, but incontinence was completely resolved in nine children (75%) [12]. Regarding the frequency of the sessions, a Brazilian study conducted by Veiga underlined that in the case of children with OAB who are unable to undergo parasacral TENS treatment three times weekly, the method can be administered successfully at twice-weekly sessions [13]. A recent meta-analysis summarizes that PTENS has demonstrated better response rates and fewer side effects compared to conventional first-line treatments, such as standard urotherapy and antimuscarinic drugs, in the case of children with OAB [14].

### 3.2. Electro-Magnetic Stimulation

Currently, electrical stimulation is combined with magnetic stimulation, resulting in a complex therapy that yielded positive results, being well accepted and tolerated by patients. This method can generate deeper stimulations when compared to electrical stimulation, which is characterized by safety and less pain compared to electrostimulation. Another important advantage of this method, especially in children, is that it can be performed while wearing clothes. Nevertheless, the method has its disadvantages, such as a longer treatment period (1–2 months) consisting of 8–12 sessions, associated difficulties in patients with a pacemaker due to its potential for causing malfunctions, and also in patients with tattoos due to the increased likelihood of burns. In spite of the above-mentioned disadvantages, it remains a suitable method in pediatric practice since these situations are less frequent when compared to adult populations.

Electro-magnetic, extracorporeal stimulation is based upon the penetration of a magnetic field through clothing into body tissues without significant alteration. The functional magnetic field is generated by a current pulse passing through a wire coil within the device’s applicator [1]. Direct stimulation of the pelvic floor muscles can be performed painlessly, without the need for insertion of an anal or vaginal probe. Recent studies reported on the utilization of the FMS Chair in women with OAB, stress urinary incontinence, and postprostatectomy urinary incontinence among males [15,16,17]. The FMS Chair has two inbuilt and adjustable applicators that can work simultaneously: the applicator on the seat stimulates the pudendal nerve, causing muscle contractions of the pelvic floor, and the back applicator produces sacral neuromodulation. Strength and endurance of pelvic floor muscles are both enhanced by FMS treatment. This procedure also assists patients in learning how to perform muscle-strengthening exercises. While in the treatment of women’s OAB, stress urinary incontinence, pelvic floor dysfunctions, and FMS gained importance; in terms of pediatric practice, only a few articles were identified.

One of the recent studies performed in the case of children presenting symptoms of OAB was conducted by Lin KN et al. The researchers used transcutaneous pelvic floor magnetic stimulation, a comfortable, less embarrassing, and very safe method, combined with pelvic floor rehabilitation, in a study group of 42 children compared with 50 children included in the control group, who received only bladder training. The authors found sacral nerve magnetic stimulation combined with bladder training to be more effective than training alone in children with OAB, improving symptoms such as bladder capacity and urination within two weeks [18].

FMS is an effective method for the treatment of primary nocturnal enuresis (PNE) in children, according to a prospective, controlled group study performed on 57 children with PNE, who were divided into two groups (control group: 29 cases, study group: 28 cases). Bladder function training, awakening treatment, and life and psychological interventions were performed in both of the groups, while the study group was treated with sacral nerve magnetic stimulation. Several parameters, such as the degree and frequency of enuresis (normal, mild, moderate, or severe), therapeutic outcomes, and bladder volume, were compared before and after treatment. Based on the results, the study highlighted that sacral nerve magnetic stimulation can reduce both the severity and frequency of enuresis, increasing bladder volume, and improving the overall treatment efficacy, supporting its promotion and clinical use [19].

Sacral magnetic stimulation can be combined with transcranial magnetic stimulation (TMS) in pediatric patients with PNE. Thus, Fouda BH et al. assessed the efficacy of TMS in a randomized study involving 40 pediatric patients who were divided into two groups: 22 children who received real magnetic stimulation (transcranial and sacral root stimulation) and 18 children who received sham stimulation. All patients were evaluated before, immediately after, and following the stimulation sessions, at one and three months post-treatment in terms of wet night frequency, QoL according to the International Consultation on Incontinence Questionnaire—Lower Urinary Tract Symptom Quality of life (ICIQ-LUTS QOL) score, and motor threshold (MT) as a measure of cortical excitability and neuronal integrity. Transcranial magnetic stimulation demonstrated alterations in cortical excitability, with significant differences observed at time points 2 and 3 (*p* = 0.044 and *p* < 0.001, respectively). Therefore, the combined approach offers a substantial reduction in the frequency of wet nights per week since significant improvement was noticed at one (*p* = 0.086) and three months (*p* < 0.001), along with no significant side effects and a better quality of life [20].

Regarding urinary incontinence, Volovets S et al. conducted a prospective randomized controlled clinical study on 75 pediatric patients (aged between 5 and 16 years), presenting with day and night urinary incontinence. They divided the patients into two groups: the study group, 39 cases who were treated with standard rehabilitation and an extracorporeal magnetic stimulation program for 21 days; and the control group, consisting of 36 patients, who did not receive extracorporeal magnetic stimulation. The study showed that the resolution of symptoms occurred in 94.8% of the cases included in the study group compared to only 25.4% in the control group. Thus, the study of Volovets S et al. highlighted the significant benefit of extracorporeal magnetic stimulation, not only in terms of reducing urinary incontinence episodes, but also in increasing micturition volume and improving quality of life. The authors concluded that perineal EMS enhances the effectiveness of rehabilitation in children with neurogenic urinary incontinence and represents a safe and promising approach for pediatric rehabilitation therapy [21].

Magnetic stimulation was also evaluated in children presenting complications after spinal cord injuries, such as detrusor overactivity, one of the most common complications. Thus, repetitive functional magnetic stimulation of the sacral nerve (100% stimulation intensity, 5 Hz, 20 min each time, five times a week) is incorporated into the rehabilitation program of detrusor overactivity and it seems to have a significant positive impact on patients’ quality of life according to the results of a prospective study that combined sacral nerve magnetic stimulation (100% stimulation intensity, 5 Hz, 20 min each time, five times a week), standard urinary management, acupuncture, and health education. The prospective study evaluated bladder compliance (bladder capacity/detrusor pressure) and pudendal nerve electromyography at baseline and at the end of the 8th week of treatment. Thus, bladder residual volume and voiding diaries were recorded weekly during the 8-week treatment period, and repeated 8 weeks after the end of treatment. The authors suggested, based on their results, that rFMS of the sacral nerve significantly improves bladder function, alleviates symptoms, and enhances quality of life [22,23].

Concerning the treatment of neurogenic bladder associated with overactive detrusor, FMS proved its efficiency among adults, reducing the number of uninhibited contractions, and significantly increasing maximum cystometric capacity along with maximum urinary flow rate [24].

Urinary tract dysfunctions represent a common feature of the clinical picture encountered in patients with autism spectrum disorder or Asperger syndrome. Moreover, antipsychotics and antidepressants prescribed for these patients often worsen micturition disorders. Therefore, screening for lower urinary tract symptoms (LUTSs) is recommended, and when indicated, treatment, especially non-pharmacological and non-invasive approaches such as functional magnetic stimulation (FMS), should be considered [25,26].

An overview of the included studies, including study design, patient characteristics, stimulation parameters, and outcomes, is presented in Table 1.

### 3.3. Quality of Life Investigation

Marciano R et al. conducted a cross-sectional study regarding the QoL and the emotional symptoms of children and adolescents with lower urinary tract dysfunctions (LUTDs), in which they included 88 patients and their parents for an interdisciplinary program that evaluated LUTDs and their impact on QoL. The authors used the Child Behavior Checklist (CBCL) for a correct prevalence of behavioral and emotional problems, and the Pediatric Quality of Life Inventory (version for parents and children) to correctly evaluate the QoL. Non-parametric correlation (Spearman test) and multiple linear regression analysis were used to evaluate the association between clinical variables and all the aspects related to QoL. According to the analysis of CBCL’s clinical scores, 56% of the patients had total behavioral problems, 55% internalizing, and 38% externalizing problems. Patients with voiding postponement had the lowest rates of total problems (*p* = 0.036) after comparing LUTDs with CBCL scores. Children and adolescents with LUTD and enuresis had an increased frequency of externalizing problems (*p* = 0.001), especially aggressive behavior (*p* = 0.013). Scores of patients with LUTD were significantly lower in all domains of QoL than normative data. A worse QoL was associated with behavioral problems in all aspects. Regression analysis showed that the CBCL school competence scale had the greatest impact on overall QoL. Thus, behavioral and social repercussions of LUTD are important and need to be addressed by specialists. The evaluation and treatment of this specific group of patients must be conducted by a multidisciplinary team in order to achieve the best results.

## 4. Discussion

### 4.1. General Discussions

Children’s voiding dysfunction is very challenging, and it might require multiple urological approaches that were developed and performed over the years. The end point is to preserve the filling and voiding functions of our patients. Caregiver education, behavioral interventions (urotherapy), hydration, timed voiding, pelvic floor training, and bowel management are recommended as first-line treatment. Alpha blockers, B3-adrenoceptor agonists, and anticholinergics are recommended as medication therapy.

Multiple methods of neurostimulation or neuromodulation are being researched to aid in pediatric micturition disorders. Posterior tibial nerve stimulation and parasacral transcutaneous electric nerve stimulation can be safely used in the case of children. Transcranial and sacral magnetic stimulation therapy serve as potential new therapy methods, with positive responses on various micturition disorders, such as nocturnal enuresis, urinary incontinence, overactive bladder, and overactive detrusor.

To maximize therapeutic success, in this population, deeper research is required to clarify the mechanisms of action and improve therapy regimens.

### 4.2. Public Health Implications

From a public health perspective, pediatric voiding dysfunction represents a frequent yet often underestimated condition, with significant implications for both affected children and healthcare systems. LUTD in childhood is associated with recurrent medical consultations, repeated diagnostic investigations, and long-term follow-up, placing a sustained burden on pediatric urology services. Beyond the direct medical impact, these disorders can negatively influence psychosocial development, school performance, and quality of life, frequently leading to anxiety, social withdrawal, and family distress. Consequently, early recognition and effective management strategies are essential not only for symptom control but also for preventing long-term sequelae and reducing healthcare utilization. Therefore, early health education of parents and older children is essential.

Management of pediatric voiding dysfunction is highly challenging. A variety of urological treatments have been developed to restore or preserve bladder function. First-line treatment focuses on education (for children and caregivers), combined with behavioral interventions. Pharmacologic options include anticholinergics, alpha blockers, and B3 adrenoceptor agonists. Neuromodulation and neurostimulation techniques are being investigated at the moment in order to support pediatric micturition disorders. Posterior tibial nerve stimulation and parasacral transcutaneous electrical nerve stimulation are already considered safe and increasingly accepted for wide use in practice. Recently, magnetic stimulation therapies, particularly functional magnetic stimulation, have gained attention as potential new approaches. These techniques already show promising therapeutic effects for certain conditions, such as nocturnal enuresis, urinary incontinence, overactive bladder, and neurogenic detrusor overactivity. However, to optimize treatment options and outcomes, further research is needed to clearly clarify the therapeutic protocols [23].

The chronic and relapsing nature of pediatric voiding dysfunction often requires prolonged treatment courses, combination therapies, and repeated adjustments of management strategies. Pharmacological treatments, while effective in selected cases, may be limited by adverse effects, suboptimal adherence, and the need for long-term administration, which further increases healthcare costs. In addition, persistent symptoms are associated with higher rates of emergency visits, hospital admissions for urinary tract infections, and advanced diagnostic procedures, all contributing to increased financial pressure on healthcare systems. Therefore, non-invasive neuromodulation techniques, such as transcutaneous electrical nerve stimulation and functional magnetic stimulation, may represent cost-effective adjuncts or alternatives by reducing symptom recurrence and treatment escalation

Importantly, the potential integration of safe, non-invasive neuromodulation therapies into standard care pathways could have a meaningful economic and organizational impact. By improving symptom control and reducing reliance on long-term pharmacotherapy or invasive interventions, these approaches may decrease follow-up intensity, hospital visits, and overall treatment costs. Moreover, home-based or outpatient neuromodulation protocols could enhance accessibility and adherence, particularly in pediatric populations. From a health policy standpoint, further high-quality studies are warranted to establish standardized protocols and cost-effectiveness analyses, which are essential for guiding reimbursement strategies and optimizing resource allocation within pediatric healthcare systems.

## 5. Conclusions

In the management of children’s voiding dysfunctions, such as overactive bladder, overactive detrusor, and nocturnal enuresis, external electrical stimulation demonstrated good efficiency. In recent years, electro-magnetic stimulation has gained importance in the management of children’s dysfunctional voiding, with good therapeutic results that are well tolerated by children. Despite a few clinical controlled trials, our review concerning these methods provides acceptable evidence for larger clinical application among the pediatric population. In the era of multiple new technologies, more multicenter studies for pediatric applications of these methods are needed in the future.

Improving the quality of life of patients and especially children is essential for a more sustainable society, especially at a time when Europe is facing a low birth rate and an aging population.

## Figures and Tables

**Table 1:** The main characteristics of the studies included in this review.

Author (Year)	Study Design	Population (*n*, Age)	LUTD Type	Stimulation Type	Stimulation Parameters	Treatment Protocol	Outcomes
De Gennaro et al. (2011) [2]	Narrative review	Pediatric population	LUTD	TENS/neuromodulation	Variable across studies	Variable	Neuromodulation was effective, heterogeneity of protocols
Coronas Soucheiron et al. (2023) [3]	Prospective multicenter	Children with OAB refractory to anticholinergics	OAB	Sacral TENS	10–20 Hz, intermittent	20 min/session, multiple weeks	Significant symptom improvement
Leao Santos et al. (2022) [4]	Prospective multicenter	Children with OAB	OAB	Parasacral TENS	Low-frequency, sensory level	20 min/session	Improved QoL and psychological outcomes
Casal-Beloy et al. (2021) [5]	Randomized controlled trial	Children with OAB	OAB	Sacral TENS vs oxybutynin	10 Hz, intermittent	Several weeks	Comparable efficacy to anticholinergics
Maternik et al. (2024) [6]	Prospective study	Pediatric patients	OAB	Parasacral TENS	10 Hz, continuous	Short- and long-term protocol	Sustained symptom improvement
Casal-Beloy et al. (2020) [7]	Prospective	Children with OAB	OAB	Home-based TENS	Individualized	Home-based sessions	Feasible and effective
Dos Reis et al. (2019) [8]	Prospective randomized	Children with LUTD	LUTD	TENS vs EMG biofeedback	10 Hz	Weekly sessions	Comparable improvement
Liu et al. (2022) [9]	RCT	Neurogenic OAB	OAB	TENS	Low-frequency	Repeated sessions	Significant urodynamic improvement
Kırlı et al. (2024) [11]	Prospective	Children with refractory overactive detrusor	Overactive detrusor	Parasacral TENS	Standard low-frequency	Multiple weeks	Symptom reduction
Kobayashi et al. (2022) [1]	Narrative	Mixed population	Pelvic floor dysfunction	Magnetic stimulation	Variable (10–50 Hz)	Variable	Improved pelvic floor function
Yamanishi et al. (2019) [12]	Randomized	Stress UI	SUI	Magnetic stimulation	10–50 Hz	Repeated sessions	Urodynamic improvement
Lin et al. (2025) [15]	Clinical study	Children with OAB	OAB	Pelvic floor magnetic stimulation	Functional magnetic pulses	Combined with training	Superior symptom control
Volovets et al. (2022) [18]	Prospective	Neurogenic UI	Neurogenic UI	Magnetic stimulation	Repetitive	Rehabilitation-based	Safe and effective
Frunda et al. (2025) [22]	Case report	Child with Asperger syndrome	LUTD	Functional magnetic stimulation	Individualized	Repeated sessions	Marked symptom improvement
Orduna-Martinez et al. (2025) [24]	Systematic review and meta- analysis	Children with neurogenic Bladder	LUTD	Electrostimulation	Variable	Variable	Conservative/rehabilitation-based
Veiga et al. (2021) [25]	Randomized clinical trial	Overactive bladder	Pediatric non-neurogenic OAB	Parasacral TENS	2 vs 3 sessions/week	10Hz	Conservative
Cheng et al. (2025) [26]	Systematic review and meta-analysis	Overactive bladder	OAB	Parasacral TENS	Variable	Variable	Conservative

## Data Availability

Not applicable.

## Abbreviations

The following abbreviations are used in this manuscript:

ES	Electrical stimulation
FMS	Functional magnetic stimulation
TENS	Transcutaneous electrical stimulation
OAB	Overactive bladder
ACHS	Anticholinergics
PLUTSS	Pediatric Lower Urinary Tract Symptoms Score
QOL	Quality of life
PNE	Primary nocturnal enuresis
SMS	Sacral nerve magnetic stimulation
